# Identifying influential spreaders in complex networks by an improved gravity model

**DOI:** 10.1038/s41598-021-01218-1

**Published:** 2021-11-12

**Authors:** Zhe Li, Xinyu Huang

**Affiliations:** 1 Software College, Shenyang University of Technology of China, Shenyang, 110870 People’s Republic of China; 2 Software College, Northeastern University of China, Shenyang, 110819 People’s Republic of China

**Keywords:** Physics, Statistical physics, thermodynamics and nonlinear dynamics

## Abstract

Identification of influential spreaders is still a challenging issue in network science. Therefore, it attracts increasing attention from both computer science and physical societies, and many algorithms to identify influential spreaders have been proposed so far. Degree centrality, as the most widely used neighborhood-based centrality, was introduced into the network world to evaluate the spreading ability of nodes. However, degree centrality always assigns too many nodes with the same value, so it leads to the problem of resolution limitation in distinguishing the real influences of these nodes, which further affects the ranking efficiency of the algorithm. The *k*-shell decomposition method also faces the same problem. In order to solve the resolution limit problem, we propose a high-resolution index combining both degree centrality and the *k*-shell decomposition method. Furthermore, based on the proposed index and the well-known gravity law, we propose an improved gravity model to measure the importance of nodes in propagation dynamics. Experiments on ten real networks show that our model outperforms most of the state-of-the-art methods. It has a better performance in terms of ranking performance as measured by the Kendall’s rank correlation, and in terms of ranking efficiency as measured by the monotonicity value.

## Introduction

Network science plays an extremely key role in many fields^1^. The heterogeneity of real networks^2^ puts forward a vital question: How to measure the importance of nodes quantitatively? An effective algorithm to identify influential spreaders may be a good answer. Identification of influential spreaders can be widely used in epidemic analysis^3,4^, rumor analysis^5^, power grid protection^6^, knowledge graph^7^, social computing^8,9^, information propagation^10^, community detection^11,12^, discovery of candidate drug targets and essential proteins^13^, discovery of important species^14,15^, and so on.

So far, most known methods merely use structural information^16^, which can be classified into neighborhood-based centralities and path-based centralities roughly. Typical representatives of neighborhood-based centralities are degree centrality^17^ (DC), *k*-shell decomposition method^18^ (KS) and H-index^19^ while typical representatives of path-based centralities are betweenness centrality^20^ (BC) and closeness centrality^21^ (CC).

Although the above methods are very classic, it is difficult to identify the vital nodes in complex networks accurately and efficiently. In order to solve this problem, many effective node ranking algorithms^22–29^ have been proposed in recent years, among which the algorithms based on gravity law seem very promising. Hence, a series of algorithms^28–40^ based on the gravity law have been proposed, and their performance is much better than the above classic methods. Typical representatives are gravity centrality^28^ (GC) and local gravity model^29^ (LGM). GC regards the *k*-shell value of a node as its mass, the shortest distance between two nodes in the network as its distance, while LGM regards the degree value of a node as its mass, and the shortest distance between two nodes as its distance. However, whether the degree or *k*-shell value is regarded as the mass, there is a shortcoming, i.e., DC and KS both assign too many nodes with the same value. So it leads to the problem of resolution limitation in distinguishing the real influences of these nodes, which further affects the ranking efficiency of the algorithm.

In this paper, in order to solve the above problem, we propose a high-resolution index combining both DC and KS. Furthermore, based on the proposed index and the well-known gravity law, we propose an improved gravity model to measure the importance of nodes in propagation dynamics. Experiments on ten real networks show that our model performs best in comparison with the above well-known state-of-the-art methods both in terms of ranking performance as measured by the Kendall’s rank correlation, and in terms of ranking efficiency as measured by the monotonicity value.

## Results

### Algorithms

**Figure 1:**
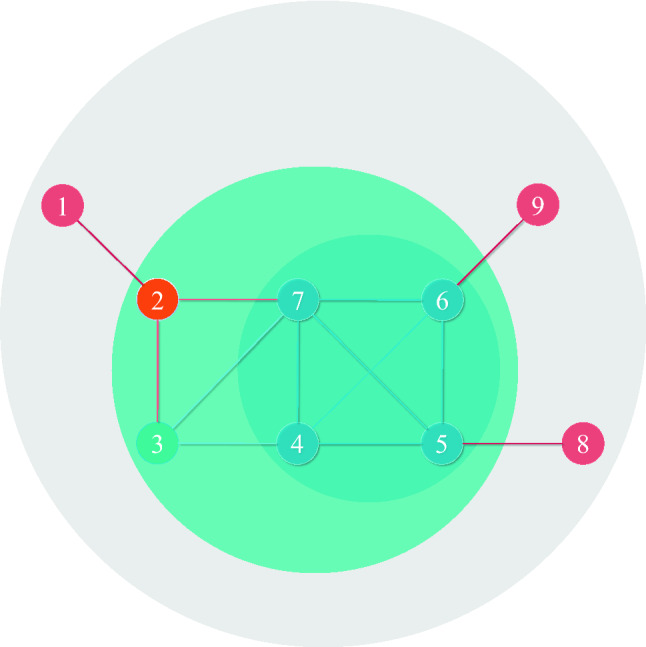
A toy network with nine nodes to illustrate the resolution limit problem for DC and KS.

Firstly, we take a toy network shown in Fig. 1 to illustrate the resolution limit problem for DC and KS. The degree and *k*-shell values of each node in the toy network are shown in Table 1. Obviously, $k(1)=k(8)=k(9)=1$, $k(2)=k(3)=3$, $k(4)=k(5)=k(6)=4$, $k_s(1)=k_s(8)=k_s(9)=1$, $k_s(2)=k_s(3)=2$, $k_s(4)=k_s(5)=k_s(6)=k_s(7)=3$, where *k*(*i*) and $k_s(i)$ are the degree and *k*-shell value of node *i*, respectively. DC and KS always assigns too many nodes with the same value, which leads to the problem of resolution limitation in distinguishing the real influences of these nodes.

**Table 1:** The degree and *k*-shell values of each node in the toy network.

Node	1	2	3	4	5	6	7	8	9
DC	1	3	3	4	4	4	5	1	1
KS	1	2	2	3	3	3	3	1	1

A simple solution is to consider both DC and KS, that is, to estimate the influence of node *i* by $k(i)+k_s(i)$. However, the problem has not been completely solved. Take node 2 and node 3 as an example, compared with node 2, node 3 is closer to the center of the network, so node 3 may be more conducive to propagation. However, we cannot distinguish the two nodes by the above proposed method. Although both node 2 and node 3 are in the 2-shell, node 3 is removed later than node 2, that is, the 2-shell decomposition process includes two stages, node 2 is removed in the first stage and node 3 is removed in the second stage. So we introduce the stage number at which the node is removed from the network while performing the *k*-shell decomposition.

Given a network *G*, during the process of *k*-shell decomposition for the *k*-degree iteration, the total number of stages is *q*(*k*), and node *i* is removed in the *p*(*i*) stage. The improved *k*-shell index of node *i* , denoted by $k_{s}^*(i)$, can be calculated by

1 $$\begin{aligned} k_{s}^*(i)=k_{s}(i)+\frac{p(i)}{\max \limits _{k}q(k)+1}. \end{aligned}$$

The process of *k*-shell decomposition and the $k_{s}^*$ value of each node in the toy network are shown in Table 2 and Table 3, respectively. Take node 3 as an example, $q(1)=1$, $q(2)=2$, $q(3)=1$, and then $\max \limits _{k}q(k)=2$, so $k_{s}^*(3)=k_{s}(3)+p(3)/(\max \limits _{k}q(k)+1)=2+2/(2+1)\approx 2.667$.

**Table 2:** The process of *k*-shell decomposition in the toy network.

Shell	Stage	1	2	3	4	5	6	7	8	9
1-shell	Stage-1	$\checkmark$							$\checkmark$	$\checkmark$
2-shell	Stage-1		$\checkmark$							
	Stage-2			$\checkmark$						
3-shell	Stage-1				$\checkmark$	$\checkmark$	$\checkmark$	$\checkmark$

**Table 3:** The $k_{s}^*$ value of each node in the toy network.

Node	1	2	3	4	5	6	7	8	9
$k_{s}^*$	1.3333	2.3333	2.6667	3.3333	3.3333	3.3333	3.3333	1.3333	1.3333

The index combining degree and *k*-shell of node *i*, denoted by *DK*(*i*), can be defined by

2 $$\begin{aligned} DK(i)=k(i)+k_{s}^*(i). \end{aligned}$$

Such index is named as degree *k*-shell (DK) index. The *DK* value of each node in the toy network are shown in Table 4. As shown in Table 4, node 2 and node 3 can be distinguished (DC, KS, DC+KS failed), node 7 can be distinguished from nodes 4–6 (KS failed), so DK index is a high-resolution index. Furthermore, DK carries both the local and global information of nodes.

**Table 4:** The *DK* value of each node in the toy network.

Node	1	2	3	4	5	6	7	8	9
*DK*	2.3333	5.3333	5.6667	7.3333	7.3333	7.3333	8.3333	2.3333	2.3333

Inspired by the gravity law, we regard *DK* value of a node as its mass and the shortest distance between two nodes in the network as their distance. Hence the influence of node *i* can be estimated as follows

3 $$\begin{aligned} DKGM(i)=\sum _{j\ne {i},d(i,j)\le R}\frac{DK(i)DK(j)}{d^{2}(i,j)}, \end{aligned}$$

where *d*(*i*, *j*) is the shortest distance from node *i* to node *j* and *R* is the truncation radius^29^. Such method is named as DK-based gravity model (DKGM). The algorithmic description of the DKGM is provided in Algorithm 1.


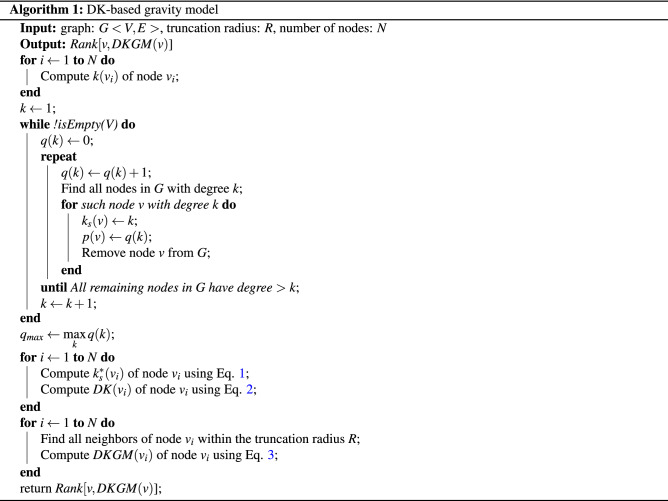


The result of DKGM with $R=2$ of the toy network is shown in Table 5. Take node 3 as an example, the 1-order neighbors of node 3 are node 2, node 4 and node 7, the 2-order neighbors of node 3 are node 1, node 5 and node 6, so $DKGM(3)=DK(3)*DK(2)+DK(3)*DK(4)+DK(3)*DK(7)+DK(3)*DK(1)/4+DK(3)*DK(5)/4+DK(3)*DK(6)/4\!\approx143.08$.

**Table 5:** The result of DKGM with $R=2$ of the toy network.

Node	1	2	3	4	5	6	7	8	9
*DKGM*	20.61	116.44	143.08	228.56	210.22	210.22	289.58	30.53	30.53

By Algorithm 1, we can find that calculating the improved *k*-shell index needs the following times operations, $N_{ks1}\left\langle k \right\rangle + N_{ks2}\left\langle k \right\rangle + \cdot + N_{ksmax}\left\langle k \right\rangle$ = $(N_{ks1} + N_{ks2} + \cdot + N_{ksmax})\left\langle k \right\rangle$ = $N \left\langle k \right\rangle$ = *M*, so the computational complexity of this part is *O*(*M*), where $N_{ks1}$ is the number of 1-shell nodes, *ksmax* is the max *k*-shell value and $\left\langle k \right\rangle$ is the average degree. The part with the highest computational complexity in our model is computing the *R*-order neighbors of each node, it needs $N \left\langle k \right\rangle ^{R}$ times operations, so the computational complexity of this part is $O(N \left\langle k \right\rangle ^{R})$. Therefore, the computational complexity of our model is $O(N \left\langle k \right\rangle ^{R})$. Fortunately, since most real networks are of small-world property, *R* is usually set to 2 or 3 to obtain the optimal result. So the computational complexity of our model in real-life applications is generally not more than $O(N \left\langle k \right\rangle ^3)$, where $\left\langle k \right\rangle \ll N$.

### Data description

In this paper, we use ten real networks from different fields to test the performance of DKGM, including four social networks (PB^41^, Facebook^42^, WV^43^ and Sex^44^), two collaboration networks (Jazz^45^ and NS^46^), one transportation network (USAir^47^), one communication network (Email^48^), one infrastructure network (Power^49^) and one technological network (Router^50^). These networks’ topological features are shown in Table 6, including the number of nodes, denoted by *N*, the number of links, denoted by *M*, the average degree, denoted by $\langle k\rangle$, the average distance, denoted by $\langle d\rangle$, the clustering coefficient^49^, denoted by *C*, the assortative coefficient^51^, denoted by *r*, the degree heterogeneity^52^, denoted by *H*, and the epidemic threshold^53^ of the SIR model^54^, denoted by $\beta _c$.

**Table 6:** The basic topological features of the ten real networks.

Networks	*N*	*M*	$\langle k\rangle$	$\langle d\rangle$	*C*	*r*	*H*	$\beta _c$
PB	1222	16714	27.3552	2.7375	0.3600	− 0.2213	2.9707	0.0125
Facebook	4039	88,234	43.6910	3.6925	0.6170	0.0636	2.4392	0.0095
WV	7115	100,762	28.3238	3.2475	0.2089	− 0.0831	5.1319	0.0069
Sex	15,810	38,540	4.8754	5.7846	0.0000	− 0.1145	5.8276	0.0365
Jazz	198	2742	27.6970	2.2350	0.6334	0.0202	1.3951	0.0266
NS	379	914	4.8232	6.0419	0.7981	− 0.0817	1.6630	0.1424
USAir	332	2126	12.8072	2.7381	0.7494	− 0.2079	3.4639	0.0231
Email	1133	5451	9.6222	3.6060	0.2540	0.0782	1.9421	0.0565
Power	4941	6594	2.6691	18.9892	0.1065	0.0035	1.4504	0.3483
Router	5022	6258	2.4922	6.4488	0.0329	− 0.1384	5.5031	0.0786

### Empirical results

**Figure 2:**
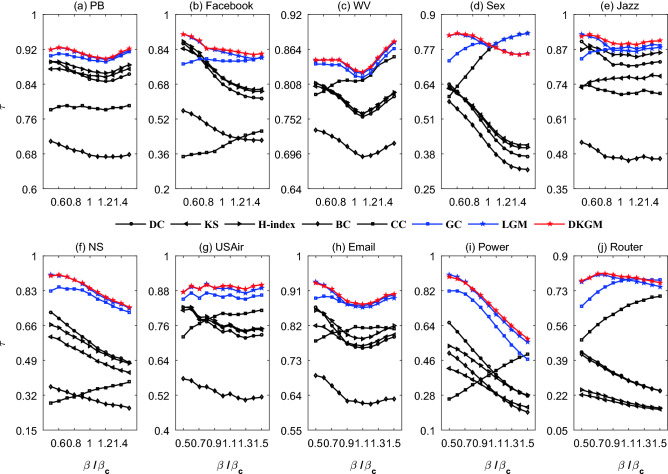
The algorithms’ accuracies measured by Kendall’s Tau for different $\beta$. The black symbols represent the five classic algorithms (DC, KS, H-index, BC and CC), the blue symbols represent the typical algorithms based on the gravity law (GC and LGM), and the red symbol represents our model.

In this paper, we apply the famous SIR model^54^ to compare the influential rankings produced by algorithms and simulations. Given the network and infection rate $\beta$, 1000 independent implementations are performed and averaged in order to obtain the standard ranking of the influences of nodes (see details about SIR model in Methods). In each implementation every node is selected once as the seed once. The accuracy of an algorithm is measured by Kendall’s Tau ($\tau$)^55^ (see details about the Kendall’s Tau in Methods) between the standard ranking and the ranking produced by the algorithm. The larger the value of $\tau$, the better the performance. The accuracies of DKGM and the seven benchmark algorithms (see details about the benchmark centralities in Methods) for $\beta =\beta _c$ are compared in Table 7, and the accuracies of different $\beta$ values are shown in Fig. 2.

**Table 7:** The algorithms’ accuracies measured by Kendall’s Tau for $\beta =\beta _c$. The parameters in the related algorithms (i.e., LGM and DKGM) are adjusted to their optimal values according to the largest $\tau$. The best algorithm for each network is emphasized by bold.

Networks	DC	KS	H-index	BC	CC	GC	LGM	DKGM
PB	0.8524	0.8595	0.8694	0.6771	0.7852	0.8948	0.9030	**0.9047**
Facebook	0.6798	0.7075	0.7066	0.4529	0.3940	0.7855	0.8275	**0.8382**
WV	0.7619	0.7657	0.7662	0.6978	0.8127	0.8216	0.8276	**0.8300**
Sex	0.4664	0.4925	0.4855	0.4118	0.7677	0.7876	0.7789	**0.7882**
Jazz	0.8150	0.7638	0.8513	0.4641	0.7008	0.8746	0.8666	**0.8892**
NS	0.5790	0.5106	0.5610	0.3003	0.3397	0.8139	0.8372	**0.8439**
USAir	0.7370	0.7529	0.7568	0.5171	0.8027	0.8583	0.8875	**0.8956**
Email	0.7653	0.7702	0.7883	0.6243	0.8163	0.8738	0.8697	**0.8779**
Power	0.4264	0.3122	0.4009	0.3254	0.3838	0.6851	0.7442	**0.7575**
Router	0.3139	0.1810	0.1928	0.3096	0.6383	0.7783	0.7894	**0.7999**

As shown in Table 7, compared with the five classic methods (DC, KS, H-index, BC, CC), GC, LGM and DKGM are very competitive. Especially in the NS, Power and Router networks, the advantage of the gravity-based methods are extremely obvious. It can be seen from Table 6 that NS, Power and Router are extremely sparse (with very few links). In this tree-like networks, there are very few cycles, that is, most paths have no alternative paths, so propagation is very difficult. In this case, neither the neighborhood-based methods (DC, KS and H-index) nor the path-based methods (BC and CC) can work well. Furthermore, compared with GC and LGM, DKGM always performs best. As shown in Figure 2, DKGM also performs very competitive compared with the seven benchmark algorithms for different $\beta$ not too far from $\beta _c$.

**Figure 3:**
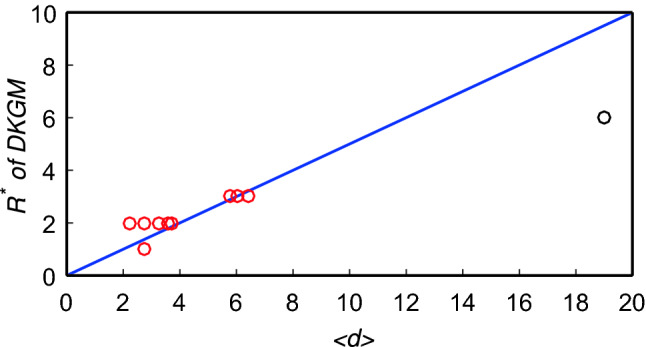
The relation between $R^*$ of DKGM and $\left\langle d \right\rangle$ for $\beta =\beta _c$. Ten circles represent ten real networks and the slope of the blue line is 1/2. The black circle is the Power network. Although the optimal truncation radius $R^*=6$ in the Power network is slightly different from what Eq. 4 predicts (i.e., $R=9$), the algorithmic accuracy at $R=9$ ($\tau =0.7366$) is very close to the best accuracy at $R^*=6$ ($\tau =0.7575$).

The optimal truncation radius $R^*$ of LGM can be estimated by

4 $$\begin{aligned} R^*\approx \frac{1}{2}\left\langle d \right\rangle \end{aligned}$$

at $\beta =\beta _c$^29^. As shown in Figure 3, DKGM still keeps this property.

Furthermore, the accuracies of GC, LGM with $R = \left\langle d \right\rangle /2$ and DKGM with $R = \left\langle d \right\rangle /2$ for $\beta =\beta _c$ are compared in Table 8. As shown in Table 8, although the truncation radius is set heuristically, DKGM still performs best among the three algorithms.

**Table 8:** The accuracies of GC, LGM ($R = \left\langle d \right\rangle /2$) and DKGM ($R = \left\langle d \right\rangle /2$) for $\beta =\beta _c$. The best algorithm for each network is emphasized by bold.

Networks	GC	LGM ($R = \left\langle d \right\rangle /2$)	DKGM ($R = \left\langle d \right\rangle /2$)
PB	0.8948	0.9030	**0.9047**
Facebook	0.7855	0.8275	**0.8382**
WV	0.8216	0.8276	**0.8300**
Sex	0.7876	0.7789	**0.7882**
Jazz	0.8746	0.8666	**0.8842**
NS	0.8139	0.8324	**0.8439**
USAir	0.8583	0.8875	**0.8904**
Email	0.8738	0.8697	**0.8779**
Power	0.6851	0.7222	**0.7366**
Router	0.7783	0.7894	**0.7999**

Finally, we apply the monotonicity^56^, denoted by $M_{r}$, to measure the ranking efficiency of algorithms. This metric is used to measure the uniqueness of the elements in a ranking list and it can be computed by

5 $$ M_{r} (L) = \left[ {1 - \frac{{\sum\nolimits_{{r \in L}} {N_{t} } (r)(N_{t} (r) - 1)}}{{N(N - 1))}}} \right]^{2}  $$

where *L* is the ranking list, and $N_{t}(r)$ is the number of ties with the same rank *r*.

The monotonicity of node ranking list produced by different algorithms is shown in Table 9. As shown in Table 9, except the PB network, DKGM always performs best among the eight algorithms. In the PB network, the reason why GC narrowly defeated DKGM is that DKGM just considers 1-order neighbors while GC considers 3-order neighbors. The results reported in Table 9 demonstrate DKGM is a remarkably high-resolution algorithm.

**Table 9:** The monotonicity of node ranking list produced by different algorithms, the best algorithm for each network is emphasized by bold.

Networks	DC	KS	H-index	BC	CC	GC	LGM	DKGM
PB	0.9328	0.9064	0.9268	0.9489	0.9980	**0.9993**	0.9991	0.9992
Facebook	0.9739	0.9419	0.9665	0.9855	0.9967	0.9998	0.9999	**0.9999**
WV	0.7761	0.7673	0.7732	0.7704	0.9994	0.9996	0.9996	**0.9996**
Sex	0.6002	0.5288	0.5457	0.6757	0.9996	0.9997	0.9997	**0.9997**
Jazz	0.9659	0.7944	0.9383	0.9885	0.9878	0.9993	0.9991	**0.9994**
NS	0.7642	0.6421	0.6825	0.3388	0.9928	0.9947	0.9933	**0.9953**
USAir	0.8586	0.8114	0.8355	0.6970	0.9892	0.9943	0.9933	**0.9951**
Email	0.8874	0.8088	0.8583	0.9400	0.9988	0.9999	0.9998	**0.9999**
Power	0.5927	0.2460	0.3930	0.8314	0.9998	0.9975	0.9999	**0.9999**
Router	0.2886	0.0691	0.0876	0.2985	0.9961	0.9962	0.9964	**0.9966**

## Discussion

Degree centrality and the *k*-shell decomposition method, as the most widely used neighborhood-based centralities, were introduced to the network world to evaluate the spreading ability of the nodes. However, the two methods always assign too many nodes with the same value, which leads to the problem of resolution limitation in distinguishing the real influences of these nodes. To solve the above problem, combining the two methods (i.e., DC and KS), we propose a high-resolution index (DK) that can simultaneously reflect the local and global information of nodes. Furthermore, we propose an improved gravity model (DKGM) that combining DK index and the gravity law to evaluate the spreading ability of nodes. The empirical results show that DKGM performs best in comparison with seven well-known benchmark methods and DKGM is a remarkably high-resolution algorithm.

A potential disadvantage of DKGM is how to set truncation radius *R*. Fortunately, as shown in Fig. 3, we find an empirical relation between $R^*$ and the average distance $\left\langle d \right\rangle$, so we can use the relation (see Eq. 4) to approximate $R^*$. In addition, since most real networks are of small-world property^49,57^, $R^*$ should be small, it can be set to 2 or 3 generally.

There are still some potential problems in the future. First of all, the original law of gravity is symmetrical, but due to the different effects of different nodes or the inherent asymmetry of dynamics^58,59^, the influence of node *i* on node *j* may be different from that of node *j* on node *i*, in which the asymmetric form of gravity law may be involved. Secondly, as the heterogeneity of the links greatly change their importance^60^, how to use gravity model in the weighted networks is still an open issue. We will also develop some other better methods based on the gravity law to identify influential spreaders.

## Methods

### Benchmark centralities

We denote an undirected and unweighted network as $G=<V,E>$, where *V* and *E* are the sets of nodes and links, respectively, denote $|V|=N$ and $|E|=M$, so the network has *N* nodes and *M* links. The adjacent matrix of *G* is represented by $A=(a_{ij})_{N\times N}$, if there is a link from node *i* to node *j*, $a_{ij}=1$, otherwise, $a_{ij}=0$.

DC^17^ of node *i* can be calculated by

6 $$\begin{aligned} DC(i)=k(i), \end{aligned}$$

where $$k(i)=\sum _j a_{ij}$$.

KS^18^ works by iterative decomposition of the network into different shells. The first step of KS is to remove all the nodes in the network whose degree $k=1$. Then it remove nodes whose degree $k \le 1$ after one round removal because this step may lead to the reduction of the degree values during the process of removal. Until there are no nodes in the network with degree $k \le 1$, all the nodes which have been removed in this step create 1-shell and their *k*-shell values are equal to one. Then repeat this process to obtain 2-shell, 3-shell, ... , and so on. Finally all nodes are divided into different shells and the *k*-shell value of each node can be obtained.

The H-index^19^ of node *i*, represented by *H*(*i*), is defined as the maximal integer value satisfying that there are at least *H*(*i*) neighbors of node *i* and degrees of these neighbors are all no less than *H*(*i*).

BC^20^ of node *i* can be calculated by

7 $$\begin{aligned} BC(i)=\sum _{s\ne {i},s\ne {t},i\ne {t}}\frac{g_{st}(i)}{g_{st}}, \end{aligned}$$

where $g_{st}$ is the number of shortest paths from node *s* to node *t*, and $g_{st}(i)$ is the number of shortest paths from node *s* to node *t* that pass through node *i*.

CC^21^ of node *i* can be calculated by

8 $$\begin{aligned} CC(i)=\frac{N-1}{\sum \limits _{j\ne i} d(i,j)}. \end{aligned}$$

GC^28^ of node *i* can be calculated by

9 $$\begin{aligned} GC(i)=\sum _{j\in \psi _i}\frac{k_s(i)k_s(j)}{d^{2}(i,j)}, \end{aligned}$$

where $\psi _i$ is the neighborhood set whose distance to node *i* is less than or equal to 3.

LGM^29^ of node *i* can be calculated by

10 $$\begin{aligned} LGM(i)=\sum _{d_{ij}\le R,j\ne {i}}\frac{k(i)k(j)}{d^{2}(i,j)}. \end{aligned}$$

### SIR model

The SIR model^54^ initially considers all nodes as susceptible (S) except the source node in the infected (I) state. Each infected node can infect its susceptible neighbors with probability $\beta$. In each subsequent step, all infected nodes change their own states to recovered (R). A node in the recovered state will never participate in the propagation dynamic process with the probability $\lambda$. The propagation process continues until there are no nodes in the infected state. The influence of node *i* can be estimated by

11 $$\begin{aligned} F(i) = N_r/N, \end{aligned}$$

where $N_r$ is the number of recovered nodes when dynamic process achieving steady state. $\lambda$ is set to 1 for simplicity, and the corresponding epidemic threshold^53^ is

12 $$\begin{aligned} \beta _c\approx \frac{\left\langle k \right\rangle }{\left\langle k^{2} \right\rangle -\left\langle k \right\rangle }, \end{aligned}$$

where $\left\langle k^{2} \right\rangle$ is the second-order moment of the degree distribution.

### The Kendall’s Tau

The Kendall’s Tau^55^ is a measure of the strength of correlation between two sequences. $$X=(x_1, x_2, ... ,x_N)$$ and $$Y=(y_1, y_2, ..., y_N)$$ are two sequences with *N* elements. For any pair of two-tuples $$(x_i,y_i)$$ and $$(x_j,y_j)$$ $$(i\ne j)$$, if $$x_i>x_j$$ and $$y_i>y_j$$ or $$x_i<x_j $$ and $$y_i<y_j$$, the pair is concordant. If $$x_i>x_j$$ and $$y_i<y_j$$ or $$x_i<x_j$$ and $$y_i>y_j$$, the pair is inconsistent. If $$x_i=x_j$$ or $$y_i=y_j$$, the pair is neither concordant nor inconsistent. Kendall’s Tau of *X* and *Y* can be defined as

13 $$\begin{aligned} \tau =\frac{2(n_+-n_-)}{N(N-1)}, \end{aligned}$$

where $n_+$ is the number of concordant pairs and $n_-$ is the number of discordant pairs.

## Data Availability

All relevant data are available at https://github.com/MLIF/Network-Data.
